# Effect of defect size and tooth anatomy in the measurements of a 3D patient monitoring tool

**DOI:** 10.1016/j.heliyon.2022.e12103

**Published:** 2022-12-06

**Authors:** Beatriz Gimenez-Gonzalez, Christof Setyo, Mikel Gomez Picaza, João Paulo Mendes Tribst

**Affiliations:** a Department of Implantology and Prosthetic Dentistry, Academic Centre for Dentistry Amsterdam (ACTA), Universiteit van Amsterdam and Vrije Universiteit Amsterdam, 1081 LA Amsterdam, the Netherlands; b Createch Medical, Polígono Kurutz-Gain Pabellón, 3B 20850 Mendaro, Spain; c Department of Oral Regenarative Medicine, Academic Centre for Dentistry Amsterdam (ACTA), Universiteit van Amsterdam and Vrije Universiteit Amsterdam, 1081 LA Amsterdam, the Netherlands

**Keywords:** Optical impression, Intraoral scanners, Complete arch, Precision

## Abstract

**Purpose:**

To assess the influence of defect size and tooth anatomy on the measurements performed by a 3D patient monitoring tool.

**Methods:**

A fully dentate model was scanned to obtain a master digital file. Virtual duplicates received defects created in molars (16, 18, 28) and incisors (11, 12, 22), according to different depths (60, 80, 120 microns) and sizes (small, medium, large) totaling 180 conditions. The surface changes measured by the 3D Patient Monitoring Tool (3Shape TRIOS Patient Monitoring [TPM]) were compared with the reference by 2 calibrated operators. False Positives (FP), and False Negatives (FN) defect were registered. Pearson chi-square test, Multivariate binary logistic regression and Spearman rank correlation were used to evaluate the data (α = 0.05).

**Results:**

A significant association was found between the area and the presence of FP and FN (P < .01). Larger defects had higher chances to present FP or an FN respectively. There was a significant association between the tooth and the presence of a FP value (FP, P = .02; FN, P = .005) specially in molars. No significant association was found between the defect depth and the presence of a FP value. Spearman rank correlation showed a strong association between the presence of an FP and an FN (r = 0.858, P < .01).

**Conclusions:**

The defect size and tooth anatomy significantly affected the virtual follow-up, whereas defect depth did not. Small defects were correctly detected in all cases. An incorrect measurement on one side of the tooth simultaneously resulted in incorrect measurement on the opposite side.

**Clinical relevance:**

The clinician should be aware that different factors related to the characteristics of the defects could affect the quality of the full-arch digital follow-up. Therefore, caution is needed when interpreting the models comparison in cases that a larger area of a tooth has been modified.

## 1 Introduction

There are innumerous advantages for restorative and prosthetic dentistry that were improved with the aid of intraoral scanners (IOs) in the digital workflow [1, 2, 3, 4, 5, 6]. Therefore, it is a consensus that digitalization make the impression process easier, less time-consuming and with lesser patient discomfort [1, 2, 3, 4, 5, 6, 7]. In addition to these know advantages, the IOs allow the digitalization of the patient anatomy, associated to the software applications to increase the potential usage in several specialties of dentistry.

As consequence, the interest and relevance of IOs increased over the years, especially in the field of prosthodontics, orthodontics, and dental implantology [[3], [6], [7], [8]]. The main applicability of IOs is to create a digital impression [2, 5]. In addition, these optical devices have been successfully used to screening periodontal issues, caries and even as a complementary tool for diagnostic assessment [3, 10]. However, the proper follow-up of tooth wear using IOs is still scarce in literature [11, 12].

The proper diagnostics of tooth wear is a challenging procedure in the dental office [13, 14]. During the aging process the teeth are in continuous effect of mechanical and/or chemical agents as an natural process of human life [15, 16]. The continuous presence of attrition and erosion are part of the tooth wear multi-factorial etiology [15, 16, 17, 18]. Therefore, tooth wear can be defined as the loss of enamel and/or dentin tissues as well as the loss of restorative materials such as dental composite, ceramics and metals caused by non-carious activity [17, 18]. Despite the physiological process caused by aging, the excessive loss of tooth structure can be pathological. The pathological tooth wear should be early identified to allow a better-informed patient and the proper treatment planning. Despite that, this disease is prevalent in 30% of children and teenagers in the Netherlands and Europe [13, 14].

Unfortunately, there is no standardized method to evaluate tooth wear in periodic follow-ups [18, 19]. Different techniques have been purposed in literature such as intraoral inspection with visual scales [27, 28] or extraoral evaluation on stone casts models. However, these methods are not qualitative, poorly accurate and subjective [19, 29, 30]. The digital profilometry could be an alternative to measure occlusal wear, but is not fully accessible with extensive time demanding and high costs, being mainly used in research [17].

Nowadays, with the aid of IOs and patient monitoring software, the assessment of surface changes in the teeth during periodic follow-up seems to be a reliable method in preventive dentistry [11, 12]. Basically, to calculate the amount of tooth wear, a baseline digital impression can be compared tooth by tooth (segmenting) with the new digital impression after a period of time. This is only possible because of the alignment between the 3D models, that can identify the dental defects generated over time [18]. Despite that, the virtual models generated by the IOs are not errors-free and the inherent limitations of the digitalization could affect how the patient monitoring tool perform the model comparison. Understanding the factors that can affect the patient follow-up with 3D tools would improve the indications and applicability of IOs and the digital patient monitoring software.

Therefore, the aim of this study was to assess the influence of digitally created defects (depth and size) and tooth anatomy on the performance of a digital follow-up tool (3Shape TRIOS Patient Monitoring [TPM]) during full-arch follow-up. The three null-hypotheses of this study were: 1) There is no significant effect of the defect size by the TPM tool follow-up; 2) There is no significant difference in detecting defects for anterior and posterior teeth; 3) There is no significant effect regarding different defect depths when using the TPM tool.

## 2 Material and methods

### 2.1 Reference model

**Figure 1:**
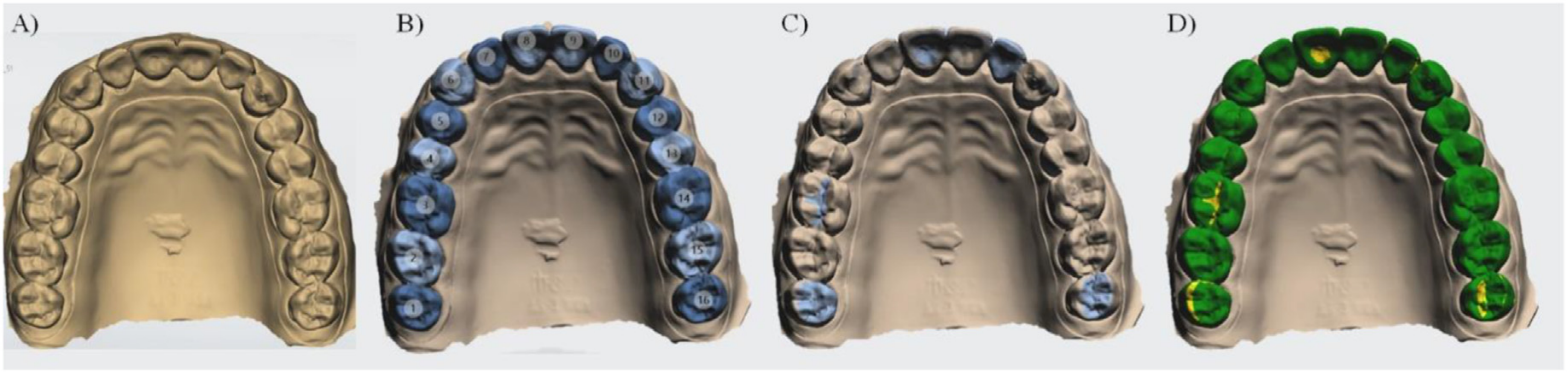
Study design: A) Master reference model, B) models superimposition for the follow-up comparison, C) major changes detected by software and D) qualitative colorimetric map generated with the models differences.

A standard language tessellation (STL) file of a fully dentated maxillary typodont model was used for the study baseline (Figure 1 A–D). For that, the digital impression was made with TRIOS intraoral scanner (3Shape, Copenhagen, Denmark) to create the “master reference model”. Then 10 duplicate models of the digital master model were created using the Computer-Aided Design (CAD) software (MeshMixer v3.5; Autodesk Inc., San Rafael, CA, USA).

### 2.2 Artificial defects

In sequence, controlled digital defects were created on the duplicates of the digital master model. For that, the CAD (MeshMixer v3.5; Autodesk Inc., San Rafael, CA, USA) was used. The duplicate models received six different defect geometries distributed according to three different size (small, medium, large) in two different tooth anatomies (Molars and Incisors) in sixty different combinations. Then, each model was replicated according to different depths (60, 80, 120 microns) totaling 180 different conditions.

**Figure 2:**
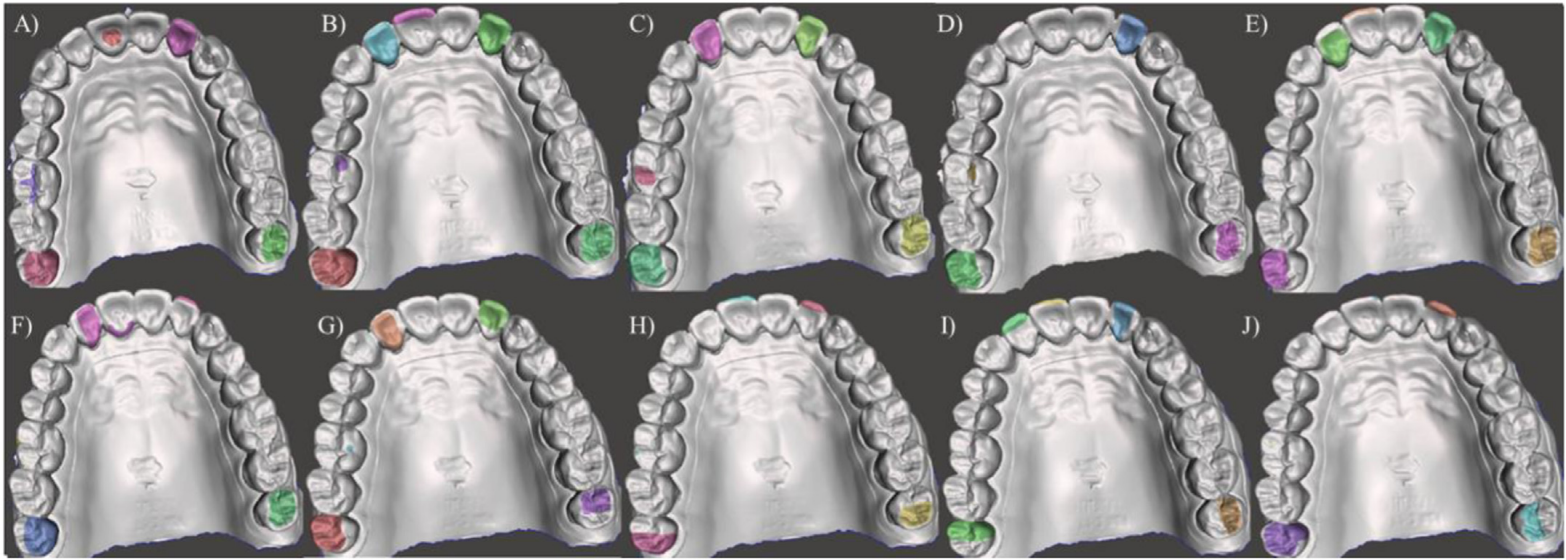
Overview of the artificial defects (Colors randomly assigned by CAD with no meaning for the results). Simulated conditions presented differently defect sizes (small, medium, large), tooth types (Incisors, Molars) according to the replica number. A) Replica 1, B) Replica 2, C) Replica 3, D) Replica 4, E) Replica 5, F) Replica 6, G) Replica 7, H) Replica 8, I) Replica 9 and J) Replica 10.

**Figure 3:**
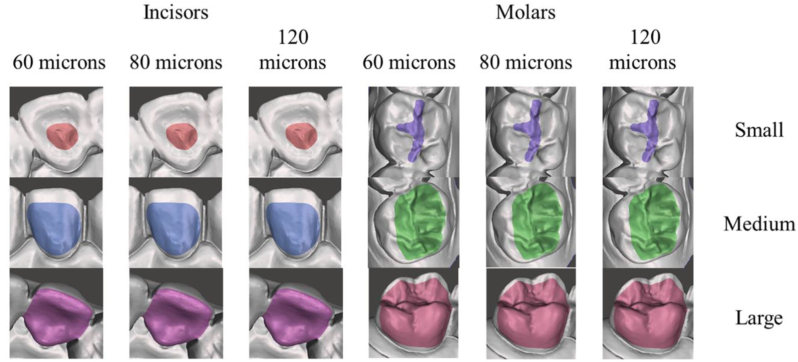
Schematic representation of the artificial defects (Colors randomly assigned by CAD with no meaning for the results) according to depth (60, 80, 120 microns) and sizes (small, medium, large) for both Incisors and Molars.

**Figure 4:**
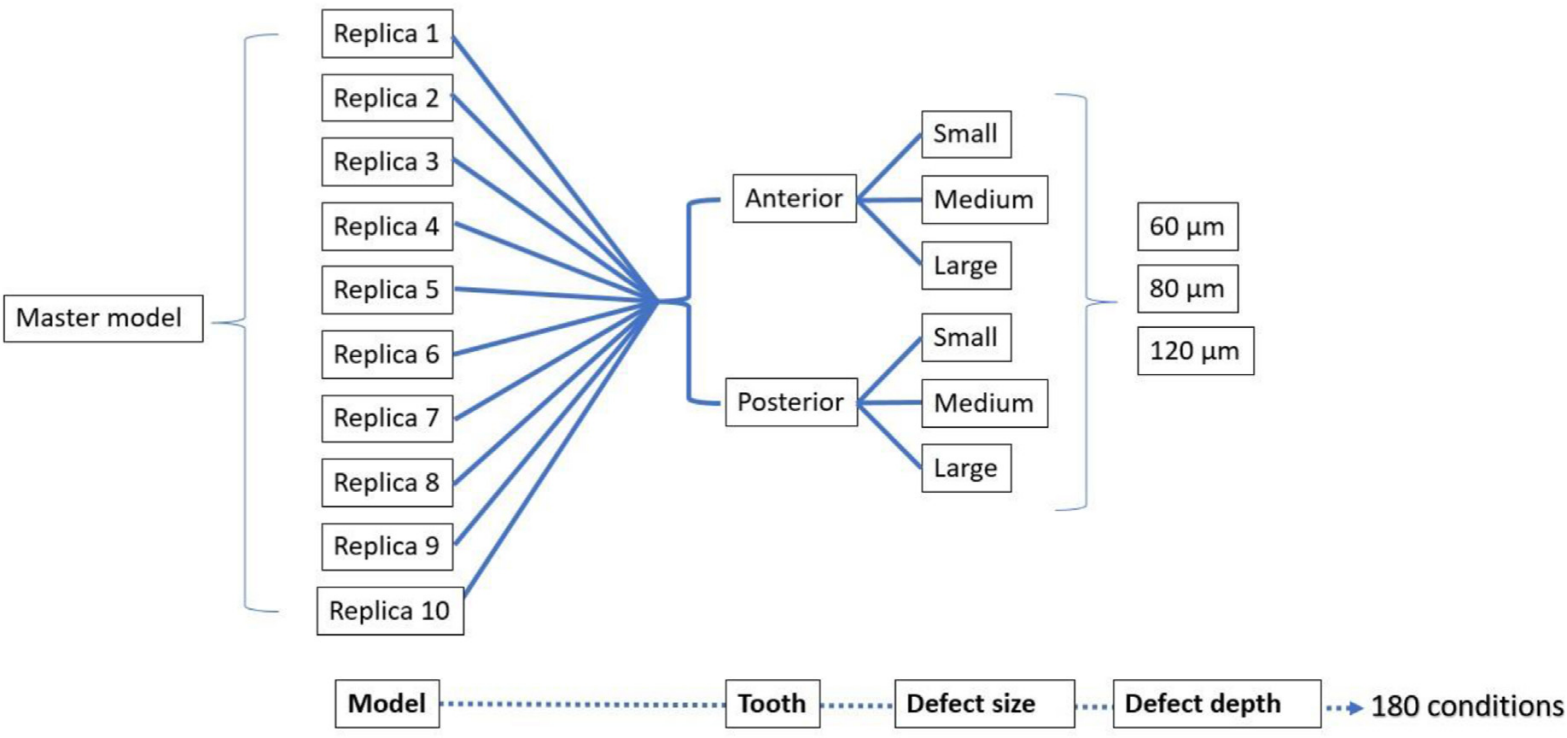
Flowchart of study design according to the artificial defects distribution in each virtual replica.

To standardize the comparison, the defect size were created manually designed (size 20, function “select”) on each digital model. For visualization, the areas of the artificial defects receive different automated assigned colors with the command “Modify” and “Create Facegroup”. Difference in defect depth was controlled by offset split using 60, 80 or 120 microns. The defects sizes were defined as follows: Small (area localized inside of the one surface of the tooth), Medium (a larger area within one surface of the tooth) and Large (substantial area affecting two surfaces of the tooth). The modified models containing the artificial defects were exported in STL. Figures 2(A–J) and 3 show an overview of the models. Figure 4 shows the study design according to the defects distribution.

To detect the differences between the control model and the replicated models, the 3D comparison tool TRIOS Patient Monitoring tool (TPM) (3Shape, Copenhagen, Denmark) was used. Two calibrated operators did the evaluation.

### 2.3 Calibration of the operators

Six models were used to train and calibrate two operators (CS and BG). The teeth defects were independently assessed by each operator. Each model was assessed at three independent moments in time, at least one week apart, to avoid recall bias. Findings were registered in SPSS, after which Cohen’s kappa was calculated to evaluate intraobserver and interobserver variability. Results of Cohen’s Kappa test varied between 0.83 and 1.0 between the different model evaluation. Therefore, a high level of agreement between the operators have been achieved for the measurements reliability.

### 2.4 Comparison of the models

For the comparison of the models, a 3D patient monitoring Tool (TRIOS Patient Monitoring Tool, 3Shape, Copenhagen, Denmark), was used. The version of the software was the 19.2.2. The master reference model and all modified models were imported to the TPM tool. The TPM tool automatically segmented the digital impression into individual teeth. No manual changes were made to the automatic segmentation. The TPM tool always compared the master reference model to 1 of the defect models using tooth by tooth superimposition. When the function “tooth comparison” was selected, the software revealed a color map of the difference between the two models. The minimum and maximum thresholds on the colorimetric scale were set to at 50 and 140 microns respectively. Either volume reduction or increase was displayed based upon the defect depth of the evaluated area. Defects between 50 and 140 microns were displayed in yellow/orange and defects above 140 microns were displayed in red.

Since the defects have been virtually defined, two outcome variables were used to classify the presence or absence of it: False Positive (FP) and False Negative (FN). A defect detected on a region where there is no created defect was considered an FP. A defect undetected on a region where there is a created defect was considered an FN. Each assessment was given a score for both FP and FN.

### 2.5 Statistical analysis

Statistical analysis was conducted using the statistical software SPSS, version 26.0 (IBM, NY, USA). Pearson chi-square test was used to study the association between one of the independent variables (tooth, defect size and depth) and the presence of an FP and/or FN. Multivariate binary logistic regression analysis was used to study the association and odds ratio between multiple independent variables. Spearman rank correlation analysis was used to study the association between the presence of FP and FN. An α of .05 was used as the cutoff point for significance.

## 3 Results

**Figure 5:**
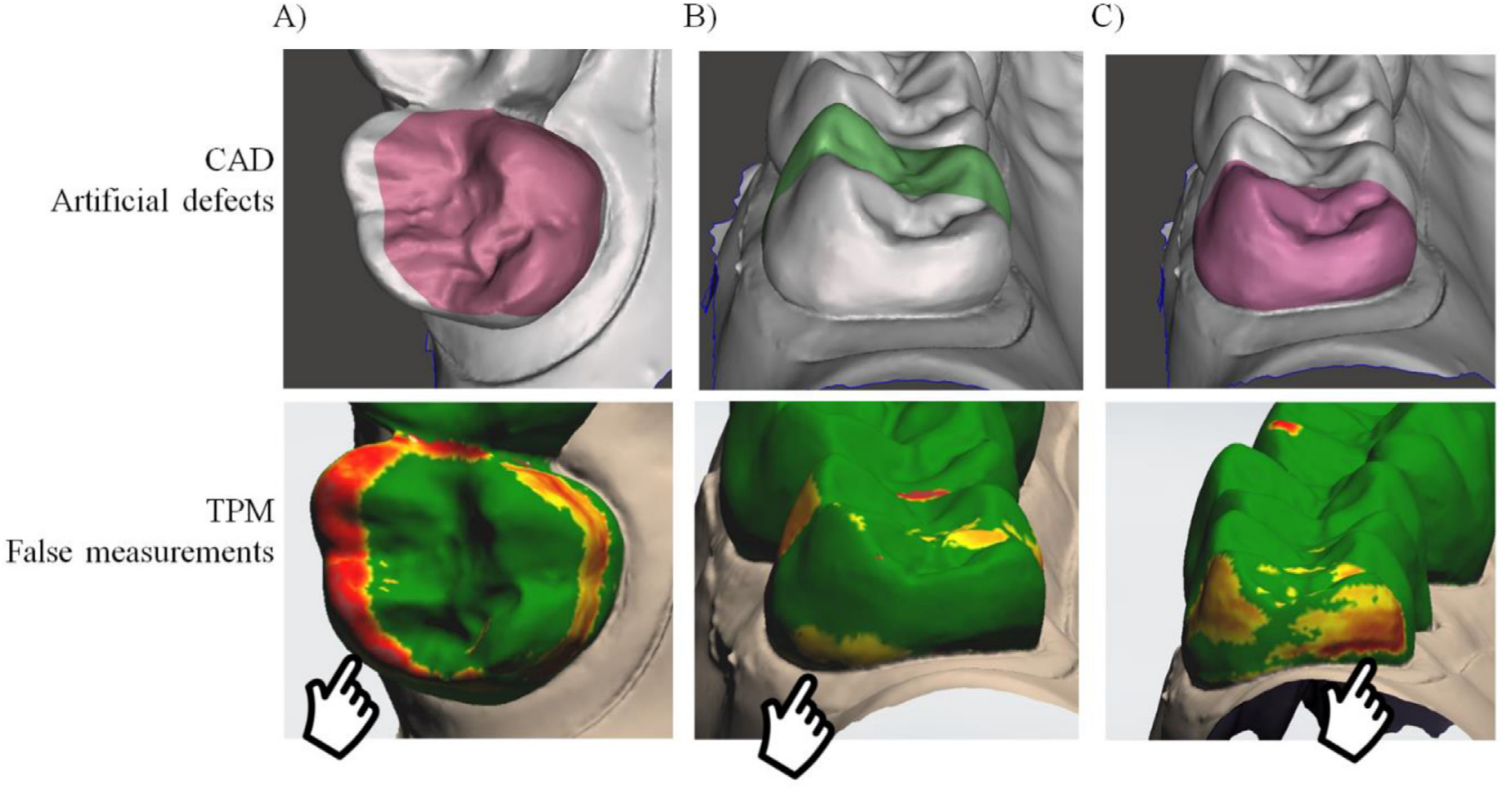
Representative colorimetric scale map showing false difference (FP and FN) values detected between to the baseline 3D model at different regions according to the artificial defects. A) Large defect in the palatal surface with false positive in the buccal surface, B) Large defect in the mesial side with false positive in the distal side of the crown and C) Large defect in the distal side with false negative detection.

**Figure 6:**
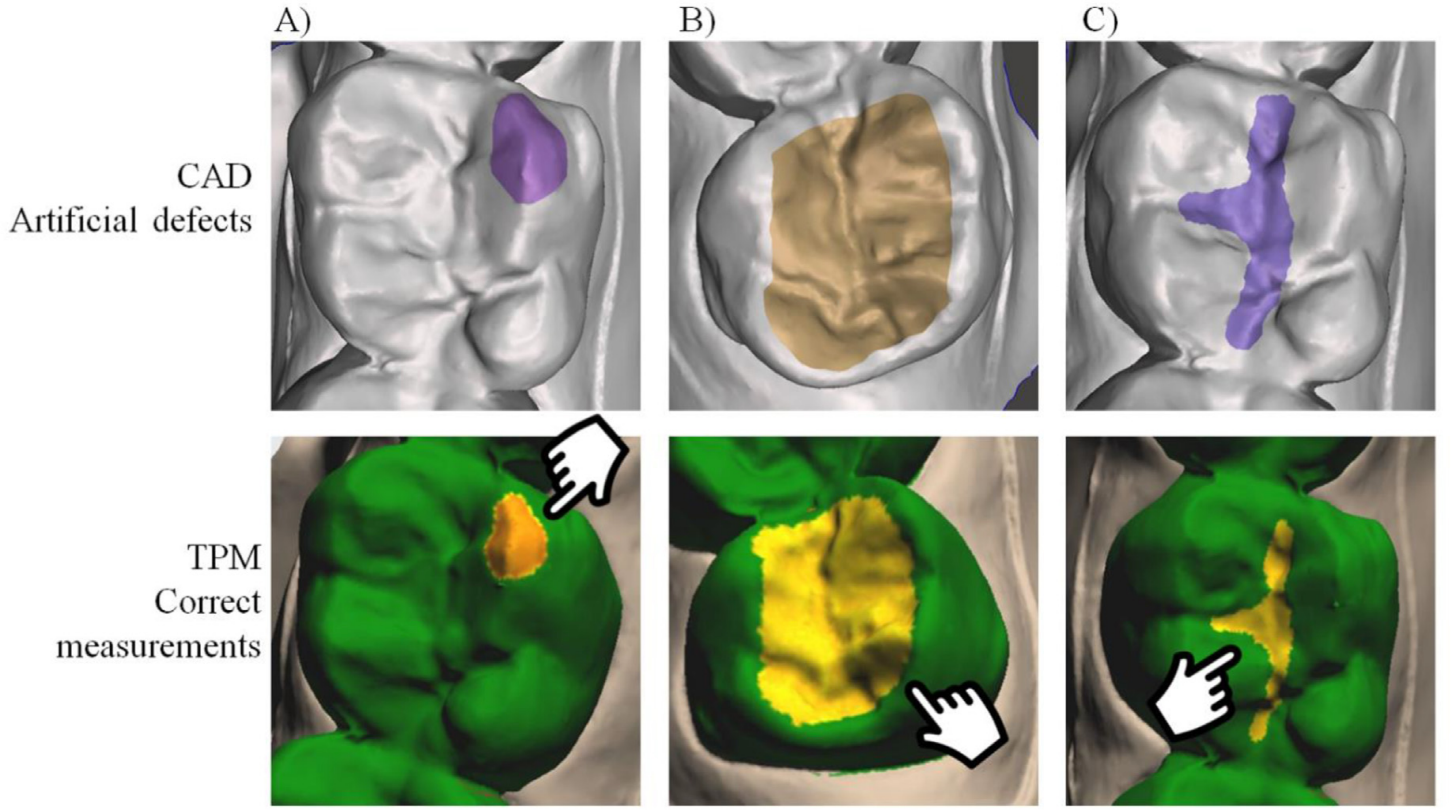
Representative colorimetric scale map showing defects detected between to the baseline 3D model at different regions according to the artificial defects. A) Small defect in the cusp with proper detection by the TPM tool, B) Medium defect in the occlusal surface with proper detection by the TPM tool and C) Small defect in the occlusal surface with proper detection by the TPM tool.

An overview of the number of FP and FN for the different defect sizes, tooth, and defect depths is presented in Table 1. Representative false defects detection can be observed in Figure 5A–C. An overall significant association was found between the defect area and the presence of an FP and/or FN (P < .01). Post hoc analysis using Fisher’s exact test was performed to assess the association between the presence of an FP and/or FN and pairs of defect sizes. Significant associations were found within all combinations of defect sizes (P < .01). A significant association was found with the presence of an FP and/or FN when the defect area became larger. No significant association was found between tooth type and the presence of an FP and/or FN. Also, no significant association was found between defect depth and the presence of an FP and/or FN. Representative defects detection can be observed in Figure 6A–C.

**Table 1:** Pearson chi-square test and the presence of an FP and/or FN in percentages.

Independent Variables (N = 60)	FP			FN			FP and FN		
	No	Yes	X^2^ (P value)	No	Yes	X^2^ (P value)	No	Yes	X^2^ (P value)
**Defect size**			102.857			111.150			111.150
Small	60 (100%)	0 (0%)	(<.01)	60 (100%)	0 (0%)	(<.01)	60 (100%)	0 (0%)	(<.01)
Medium	52 (87%)	8 (13%)		51 (85%)	9 (15%)		51 (85%)	9 (15%)	
Large	12 (20%)	48 (80%)		9 (15%)	51 (85%)		9 (15%)	51 (85%)	
**Tooth**			2.592			3.600			3.600
Incisor	67 (74%)	23 (26%)	(.11)	66 (73%)	24 (27%)	(.06)	66 (73%)	24 (27%)	(.06)
Molar	57 (63%)	33 (37%)		54 (60%)	36 (40%)		54 (60%)	36 (40%)	
**Defect depth**			0.674			0.150			0.150
60 microns	43 (72%)	17 (28%)	(.71)	40 (67%)	20 (33%)	(.93)	40 (67%)	20 (33%)	(.93)
80 microns	42 (70%)	18 (30%)		41 (68%)	19 (32%)		41 (68%)	19 (32%)	
120 microns	39 (65%)	21 (35%)		39 (65%)	21 (35%)		39 (65%)	21 (35%)	

X^2^ and P values from the Pearson chi-square test are given to show the significance when comparing one independent variable (defect size, tooth type, defect depth) to the outcome variable (PF and/or FN).

Since small defects have been correctly detected (Table 1), only medium and large defect sizes were included in the multivariate binary logistic regression analysis. Results are shown in Table 2. Both defect area (P < .01) and tooth type (P = .02) showed a significant association with FP, while defect depth showed no significant association with FP.

**Table 2:** Multivariate binary logistic regression analysis for FP and/or FN. Coefficient, standard error, P value, odds ratio, and the confidence interval of the multivariate binary logistic regression analysis.

Independent variables	FP			FN (identical to FP and FN)		
	B (SE)	P value	OR (95%CI)	B (SE)	P value	OR (95%CI)
**Defect area**						
Medium	Reference			Reference		
Large	3.61 (0.58)	<.01	37.10 (11.96–115.11)	4.064 (0.67)	<.01	58.227 (15.75–215.3)
**Tooth type**						
Incisor	Reference			Reference		
Molar	1.33 (0.56)	.02	3.77 (1.26–11.32)	1.852 (0.66)	.005	6.372 (1.73–23.42)
**Defect depth**						
60 microns	Reference	.44		Reference	.81	
80 microns	0.19 (0.62)	.76	1.21 (0.36–4.12)	-0.214 (0.66)	.74	0.807 (0.224–2.914)
120 microns	0.78 (0.64)	.22	2.18 (0.63–7.59)	0.214 (0.66)	.74	1.238 (0.343–4.470)

As for the defect area, large defects had 37.1 times higher odds to have FP than medium defects when adjusted for tooth type and defect depth. As for tooth type, molars had 3.8 times higher odds to have FP than incisors when adjusted for defect area and defect depth. Both defect area (P < .01) and tooth type (P = .005) were significantly associated with FN, while defect depth was not significantly associated with FN in the multivariate binary logistic regression analysis.

In addition, large defects had 58.3 times higher odds to have FN than medium defects when adjusted for tooth type and defect depth. As for tooth type, molars had 6.4 times higher odds to have FN than incisors when adjusted for defect area and defect depth. Results in the multivariate binary logistic regression analysis for the combination of FP and FN were identical to the results for FN.

Spearman rank correlation analysis was used to study the association between the presence of FP and FN. The Spearman rank correlation analysis showed a strong association (r = 0.858; P < .01) between the simultaneous presence of an FP and an FN. Meaning, an assessed defect containing an FP mostly resulted in an FN also being present.

## 4 Discussion

The present study aimed to assess the influence of digitally created defects depths, area and size on the performance of a digital follow-up tool. Based on the present results, the first null hypothesis was rejected, since there was a significant association between the defect area and the values detected by the TPM tool. The second null-hypothesis was accepted, once there was no significant effect of defect depth and the values detected by the TPM tool. The third null-hypothesis was partially rejected. Pearson chi-square test did not show a significant effect regarding tooth type and the defect detected by the TPM. On the contrary, the multivariate binary logistic regression analysis showed a significant effect between the defects detected by the TPM tool and tooth type.

The TPM detected all small defects with precision, namely the presence of any FP and/or FN. Small defects were not included in the multivariate binary logistic regression analysis, because the odds ratio could not be calculated when there were zero recurrences of false values. The present study showed that the factor defect area was significantly associated with an incorrect measurement. One possible explanation could be the “best fit alignment” during the superimposition of the models [9, 10]. As the TPM overlapped two segmented scans of the same tooth obtained at two different points in time, to perform the overlap, the software assumed that the two geometries (teeth) are equal [19, 20, 21]. Therefore, when a large defect area was present, the software might move the geometries to compensate that deviation, performing an average superimposition. This approach would generate new defects (FP) reducing the detection of real defects (FN). This could also explain why small defects were correctly detected, since the portion of matching surfaces was nearly similar.

A previous report used IOs and then applied the TPM tool to assess tooth tissue loss [19]. For that, scans of a partial model were made as baseline. Then the subjects were exposed to acid and mechanical stress with virtual impressions in between the artificial aging process. The authors reported promising results regarding the capability of TPM tool to assess tooth wear. Different from the present study, the authors compared the models as a whole, instead of the tooth by tooth comparison. In reason of that, the target areas have to be manually selected in the software to compare two models. In the current study the manufacturer’s indicated option was used to assess the tooth wear using tooth by tooth comparison in an automated process. The automated approach is suggested since in a clinical situation, it is not always possible to know which areas remained stable and which areas suffered any modification. In addition, the accumulation of error for full-arch impressions is generally greater than partial arch, justifying the difference in values between both studies [3, 4, 20, 21, 22].

An *in-vitro* investigation evaluated the applicability of TPM tool to monitor erosive tooth wear using different alignment software with distinct quantitative measurement metrics [9]. For that, the authors superimposed the virtual models of bovine crowns before and after acid exposure. According to them, the IOs is a potential tool to detect and monitor early and advanced erosive tooth wear [9]. The present study corroborates that statement since the TPM tool was able to identify several defects in the models, in addition the present investigation complement the previous findings suggesting that large defects should be carefully evaluated to avoid misunderstandings during the wear measurement.

In addition to the wear measurement, the TPM tool has been reported as a complementary tool to driven tooth preparation [10]. According to a previous report, this software can be applied to control the preparation procedure of natural teeth for ceramic veneers, crowns, or fixed partial dentures. In summary, it can compare the baseline model with the prepared model, checking regions that requires more tissue removal [10]. However, it is important to guarantee the precise superimposition of the scans; and the preparation procedure should start from a preoperative scan or a trial restoration scan [10]. The present study is in agreement with that, since old models can present innumerous modified areas in comparison with the current patient condition, and the compensation during the alignment of the models could generate errors in the preparation spot. In addition, attention should be given when several teeth are being evaluated, reducing the number references faces for a proper alignment and increasing the errors chances as demonstrated in the present study when large defects were investigated.

A clinical trial evaluated the tooth wear in young adults, using IOs and digital monitoring of the mandibular first molar over 12 months [11]. The authors observed a median loss values of 31–43 μm at cusps. Similar to the present study, the authors applied Best-fit alignment for superimposition of the models, however the reference region was not informed. According to the authors, discussions are needed regarding the accuracy of IOs to display tissue loss in the complete dentition at the micro level [11]. The present study, can start this discussion showing that the full-arch follow-up using digital tools can be properly performed, if the factors that affect the models superimposition are known by the operator.

Another investigation evaluated the sensitivity of the IOs for measuring dental wear (6 and 12 months follow-up) as well as the patients' satisfaction with the use of the scanner [12]. According to their findings, the IOs showed promising levels of sensitivity to be applied as a diagnostic tool for tooth wear assessment, while the patient satisfaction was positive in all cases [12]. As a complementary investigation, the present study corroborates with the literature affirming that the use of IOs is useful and can provide an methodological procedure for the wear assessment in daily clinics [10, 11, 12]. However, the proper follow-up should be made considering the errors as well as the factors that affect the digital tool during wear detection. However, there is lack of data in literature evaluating the factors that affect the digital patient motoring by 3D superimposition and further studies are required to deeply assess the errors during dental follow-up.

It is important to consider the parameter used to display dental defects in the software [25]. In the software version used in the study, no distinction can be made between the reduction (negative) or gain (positive) in volume. This could lead to misunderstanding of the data by the clinician. Another limitation is that the software only displays defects greater than 50 microns, as the minimum threshold established by the manufacturer. The averages tooth wear per year is 30 microns although the actual rate in the dental practice was expected to be higher [23, 24, 25, 26]. Given the minimum 50 microns and the average yearly rate of tooth wear, the TPM could offer useful information in two years follow-up. Furthermore, the TPM segmentation can be indicated to optimize the quality of the comparison and to reduce the errors accumulation in full-arch comparison [22]. However, as possible consequence of segmentation could be that tooth migration could not be evaluated, when full-arch 3D models are not compared as a whole [27, 28].

A previous in vivo study performed an in vitro validation to assess four different algorithms implemented in the IOS system for automated caries detection. Similar to the present study, the authors used the TPM tool for that [29]. According to their findings, the IOs system exhibits promising performance for clinical application on occlusal changes detection and different approaches can be investigated for possible optimization of the system [29]. The present investigation suggest the tooth segmentation as one possible parameter for that, reducing the full-arch error distortion during the patient follow-up.

The findings of the present study bring up new questions regarding the factors affecting the TPM tool. A question that remains unsolved would be from which point a change in the maxillary arch significantly starts to affect the software detection. An example could be to measure the percentage of overall volume loss of the tooth. A possible next step in research could be to study volumetric assessments associated with other factors that affect TPM tool measurement as well as the wear detection in different restorative materials [30, 31, 32]. Additionally, another variable to consider would be the anatomy of each defect and the possible differences between the TPM tool and other digital patient monitoring software.

There are inherent study’s limitations that need to be taken into account before a clinical extrapolation. The *in vitro* environment simulated herein represents a different scenario than a clinical evaluation [25, 26, 27, 28]. Additionally, to calculate the wear, only the highest value of each area was selected and not the total deviation. Therefore the average wear was not truly characterized by these values. Since the tool does not distinguish between positive and negative deviations some values can be considered underestimated [4]. Based on that, it is recommended to compare the groups and to interpret the results considering the previous reported publications and other further studies to confirm or not the findings presented here.

According to the results, the clinician should be aware that different factors inherent to the evaluated patient could affect the detection of dental defects. The software seems reliable in detecting small defects, such as chipping, early-stage erosion, attrition, or abrasion. However, incorrect values are expected when defect areas became larger, due to more extensive erosion or longer periods of follow-up. Caution is needed when interpreting the TPM tool colorimetric maps in case with multiples and large defects are present.

## 5 Conclusions

Within the limitations of the present study, it is possible to conclude that the defect size and tooth anatomy significantly affected the follow-up, whereas defect depth did not. Small defects were correctly detected in all cases. An incorrect measurement on one side of a tooth simultaneously resulted in incorrect measurement on the opposite side.

## Declarations

### Author contribution statement

Beatriz Gimenez-Gonzalez: Conceived and designed the experiments; Performed the experiments; Analyzed and interpreted the data; materials, analysis tools or data; Wrote the paper.

Christof Setyo: Performed the experiments; Analyzed and interpreted the data; Wrote the paper.

Mikel Gomez Picaza: Conceived and designed the experiments; Analyzed and interpreted the data; Wrote the paper.

João Paulo Mendes Tribst: Analyzed and interpreted the data; materials, analysis tools or data; Wrote the paper.

### Funding statement

This research did not receive any specific grant from funding agencies in the public, commercial, or not-for-profit sectors.

### Data availability statement

Data will be made available on request.

### Declaration of interest’s statement

The authors declare no conflict of interest.

### Additional information

No additional information is available for this paper.
